# Notoginsenoside Fc ameliorates renal tubular injury and mitochondrial damage in acetaminophen-induced acute kidney injury partly by regulating SIRT3/SOD2 pathway

**DOI:** 10.3389/fmed.2022.1055252

**Published:** 2023-01-06

**Authors:** Miaomiao Wei, Yuancheng Gao, Dongsheng Cheng, Haiying Zhang, Wei Zhang, Yilan Shen, Qunwei Huang, Xiaoning An, Bing Wang, Zhonghai Yu, Niansong Wang, Hongbo Chen, Youhua Xu, Dingkun Gui

**Affiliations:** ^1^College of Fisheries and Life Science, Shanghai Ocean University, Shanghai, China; ^2^Department of Nephrology, Shanghai Sixth People's Hospital Affiliated to Shanghai Jiao Tong University School of Medicine, Shanghai, China; ^3^The Third Affiliated Clinical Medical College, Zhejiang Chinese Medical University, Hangzhou, China; ^4^Department of Nephrology, Shanghai Yangpu Hospital of Traditional Chinese Medicine, Shanghai, China; ^5^Department of Traditional Chinese Medicine, Shanghai Sixth People's Hospital Affiliated to Shanghai Jiao Tong University School of Medicine, Shanghai, China; ^6^Department of Nephrology, The First Affiliated Hospital of Zhejiang Chinese Medical University (Zhejiang Provincial Hospital of Traditional Chinese Medicine), Hangzhou, China; ^7^Faculty of Chinese Medicine, State Key Laboratory of Quality Research in Chinese Medicine, Macau University of Science and Technology, Macao, China; ^8^Department of Central Laboratory, Shanghai Sixth People's Hospital Affiliated to Shanghai Jiao Tong University School of Medicine, Shanghai, China

**Keywords:** acetaminophen, acute kidney injury, notoginsenoside Fc, mitochondria dysfunction, SIRT3/SOD2

## Abstract

**Introduction:**

Mitochondria dysfunction is one of the primary causes of tubular injury in acute kidney injury (AKI). Notoginsenoside Fc (Fc), a new saponin isolated from Panax notoginseng, exhibited numerous pharmacological actions. However, the beneficial effects of Fc on renal tubular impairment and mitochondrial dysfunction in AKI have not been fully studied.

**Methods:**

In this study, we established acetaminophen (APAP)-induced AKI model in mice to examine the therapeutic impacts of Fc on AKI.

**Results:**

Our results showed that Fc could decrease the levels of the serum creatinine (Scr), blood urea nitrogen (BUN) and Cystatin C in mice with AKI. Fc also ameliorated renal histopathology, renal tubular cells apoptosis and restored expression of apoptosis-related proteins such as Bax, Bcl-2 and caspase3 (C-caspase3). Additionally, Fc increased the protein expression of SIRT3 and SOD2 in kidneys from mice with AKI. *In vitro* studies further showed Fc reduced the apoptosis of HK-2 cells exposure to APAP, attenuated the loss of mitochondrial membrane potential and decreased the formation of mitochondrial superoxide. Fc also partly restored the protein expression of Bax, Bcl-2, C-Caspase3, SIRT3, and SOD2 in HK-2 cells exposure to APAP.

**Conclusion:**

In summary, Fc might reduce renal tubular injury and mitochondrial dysfunction in AKI partly through the regulation of SIRT3/SOD2 pathway.

## Introduction

Acute kidney injury (AKI) is very common in intensive care unit (ICU) and is independently related to high mortality and morbidity. Non-steroidal anti-inflammatory drugs (NSAIDs) are important causes of AKI. Acetaminophen (APAP), one of NSAIDs, is the most commonly used drug for the treatment of pain and fever. The poisonous metabolite N-acetyl-P-benzoquinone-imine (NAPQI), which is generated by APAP metabolism by cytochrome P-450 isoenzymes present in the kidney can lead to APAP-induced renal tubular injury. When taken in excess, APAP can cause severe hepatotoxicity, which is common when taken as an analgesic and antipyretic (1, 2). Numerous studies have suggested that several interrelated factors, such as the generation of reactive oxygen species (ROS), hemodynamic abnormalities, adverse effects of APAP, and inflammatory renal injury, contributed to APAP-induced nephrotoxicity, which resulted in kidney damage (3–6). N-acetyl-L-cysteine (NAC), an unspecific remedy that replenishes endogenous GSH, is an available clinical treatment for APAP overdose (7). NAC, however, needs to be given as soon as possible due to its very brief therapeutic time window (8, 9). Therefore, there is an urgent need for the development of novel and definitely effective approaches for preventing or treating APAP-induced AKI.

Notoginsenoside Fc (Fc) is a novel saponin isolated from Panax notoginseng and exhibited numerous pharmacological actions. It was reported that Fc effectively inhibited platelet aggregation (10). Fc has been demonstrated to produce many pharmacological effects (11, 12). One recent report demonstrated Fc accelerated re-endothelialization following vascular injury in diabetic rats through promoting autophagy (13). Fc could ameliorate vascular endothelial cell injury by regulation of PPAR-γ-mediated pathway in diabetic rats (14).

Our previous study found that Xuesaitong (XST), consisting of total saponins from Panax notoginseng attenuated podocyte apoptosis in streptozotocin-induced diabetic rats (15). However, the protective effects of Fc on renal tubular injury and mitochondrial dysfunction in AKI have not been fully investigated yet. This study aimed to test the hypothesis that Fc could attenuate renal tubular injury and mitochondrial damage in APAP-induced AKI and then provide novel therapy for AKI.

## Materials and methods

### Drugs preparation

APAP and NAC were purchased from MedChemExpress (Shanghai, China). Notoginsenoside Fc (purity ≥ 98%) was purchased from Shanghai Jingke Chemical Technology Co., Ltd. (Shanghai, China).

### Animal studies

All the animal procedures were conducted in accordance with the “Guide for the Care and Use of Laboratory Animals” published by the National Institutes of Health. All the experimental protocols were approved by the Animal Ethics Committee of Shanghai Jiao Tong University Affiliated Sixth People's Hospital. 6-week-old male C57BL/6 mice, weighing 18 ± 2 g, were obtained from Nanjing University's Model Animal Research Center and housed in a specified pathogen-free (SPF) environment. Five mice were housed in each separately ventilated cage, which was kept in a setting free of pathogens. A 12 h/12 h light cycle and temperature control were provided in the housing. After acclimating to the experimental region for seven days, 48 male mice were randomly divided into six groups: Control group (CTR, *n* = 8), APAP group (500 mg/kg/d, intraperitoneally (i.p). *n* = 8), APAP + Fc group (low-dose Fc, 2.5 mg/kg/d, intragastric administration (i.g). *n* = 8), APAP + Fc group (medium-dose Fc group, 5 mg/kg/d, i.g. *n* = 8), APAP + Fc group (high-dose Fc group, 10 mg/kg/d, i.g., *n* = 8), APAP + NAC group (150 mg/kg body weight, i.p. *n* = 8). Fc or NAC was administrated to mice from day 1 to day 7 and APAP was administrated to mice from day 5 to day 7. An identical volume of normal saline was intraperitoneally given to control mice.

### Renal function analysis

To assess whether Fc improves renal function in APAP-induced acute kidney injury, blood samples were centrifuged at 12,000 rpm for 20 min at 4°C. Blood supernatants were used to assess serum creatinine (Scr), blood urea nitrogen (BUN), blood aspartate aminotransferase (AST) and alanine aminotransferase (ALT), the above kits were purchased from Nanjing Jiancheng Bioengineering Institute (Nanjing, China). ELISA kit for cystatin C was purchased from BOSTER (Wuhan, China). Kidney weight and body weight were also measured.

### Transmission electron microscopy studies

The mitochondria in tubular cells of renal tissues were examined by transmission electron microscopy (TEM). Briefly, Pieces of renal cortex were dehydrated in graded ethanol, fixed with 2.5 percent glutaraldehyde in 0.1 M phosphate buffer (pH 7.4, 4°C) for 4 h, post-fixed in 1 percent phosphate-buffered osmium tetroxide for 2 h, and then embedded in Epon resin using an EMbed-812 embedding kit (SUPPLIES, PA, USA). With the aid of a JEM-2200 FS transmission electron microscope (HITACHI, Tokyo, Japan), sections (60–80 nm) were positioned on copper grids, stained with uranyl acetate, and examined afterward.

### Renal histological studies

Following anesthesia, intraperitoneal injections of 5 percent chloral hydrate were used to put all the animals to sleep. After being fixed for 48 h at room temperature in 10% neutral-buffered formalin, renal tissues were dried, embedded in paraffin, and sectioned at a thickness of 4 μm. Kidney tissue was collected and stained with hematoxylin-eosin (H&E), and periodic acid Schiff (PAS). In addition, renal histopathological lesions were observed under an inverted microscope and pathological images were randomly collected. The tubular injury score (cell necrosis, loss of brush borer, cast formation, and tubule dilation) was graded and scored from 0 to 5 as follows: 0, none; 1, 10%; 2, 11% to 25%; 3, 26% to 45%; 4, 46% to 75%; and 5,76% (16).

### TUNEL assay

Kidney cell apoptosis was detected by utilizing an Apoptosis Detection Kit (Merck, United States) on 4 μm frozen slices of kidney samples. Cells were quickly treated with 4 percent paraformaldehyde and given two 10 min PBS washes. PBS containing 0.5 % Triton X-100 was incubated on the slices for 5 min at room temperature. The TUNEL solution was then added after washing the sections twice with PBS. DAPI was used as a counterstained for nuclei. A fluorescent microscope was used to capture the images (Leica, Germany).

### Immunohistochemistry

At 7 days after administration, mouse kidney tissue was collected and immediately fixed in 4% polyformaldehyde. It was then dehydrated and sliced. Samples were immunostained with SIRT3 antibody, Ac-SOD2 antibody, and anti-SOD2 antibody, then stained with horseradish peroxidase combined with anti-Rabbit IgG polymer, and finally stained with the chromogenic agent to show representative histological micrographs.

### Cell culture

Human renal tubular epithelial cells (HK-2 cells) were grown in DMEM/F12 medium containing 10 percent fetal bovine serum. When cells achieved 70–80% confluency, they were trypsinized and passaged. Six groups were formed from logarithmic phase cells: (1) CTR, cells cultured in DMEM/F12 media; (3) APAP, cells cultured in 10 mM APAP; (4) Fc-L, cells cultured in 10mM APAP + 1μM Fc; (4) Fc-M, cells cultured in 10 mM APAP +5μM Fc; (5) Fc-H, cells cultured in 10mM APAP +10 μM Fc; (6) NAC, cells cultured in 10 mM APAP + 5 mM NAC. The treatment period for each group was 24 h.

### Flow cytometry

The Annexin V-FITC/PI Apoptosis Detection Kit (BD, United States) was used to measure apoptosis in HK-2 cells. Cells were trypsinized and recovered in PBS after centrifugation. Cells were once more centrifuged and then resuspended in binding buffer. Flow cytometry (Beckman Coulter, United States) was used to analyze cell suspension.

### ROS detection

The levels of ROS in HK-2 cells and renal tissue were measured by the dye Mitosox Red (Invitrogen, USA). In 6-well plates, HK-2 cells were grown and given an overnight incubation in DMEM/F12 containing 10% FBS. After pretreatment with 1, 5, 10 μM Fc, or 5 mM NAC for 2 h, HK-2 cells were grown and cocultured with APAP (10 mM) overnight. The following 20 min saw the addition of HK-2 cells together with Dye Mitosox Red (10 uM) grown in serum-free DMEM/F12. Additionally, Mitosox Red (10 uM) treatment was used to stain kidney specimens for 20 min. Under a confocal microscope, the intensity of Mitosox Red fluorescence in HK-2 cells and kidney tissues was found.

### Mitochondrial membrane potential detection

Using a JC-1 dye kit (Beyotime Tech, China), mitochondrial membrane potential was found in HK-2 cells and renal tissues. Following a 2-h pretreatment with Fc (1, 5, or 10μM) or NAC (5 mM) on HK-2 cells, APAP was used to trigger the cells overnight (10 mM). After that, HK-2 cells were grown with JC-1 detection solution for 30 min. Additionally, kidney samples were exposed to the JC-1 working solution for 30 min. Confocal microscopy was utilized to evaluate the fluorescence intensity in HK-2 cells and kidney tissues.

### Western blot

Total protein preparations from the mice kidney tissues were subjected to a Western blot. Primary antibodies of SIRT3 (1:1,000, CST, USA), SOD2 (1:1,000, ABclonal, China), Ac-SOD2 (1:1,000, Abcam, USA), Bax (1:1,000, CST, USA), Bcl-2 (1:1,000, Abcam, USA), Cleaved-Caspase3 (1:1,000, CST, USA), β-actin (1:1,000, CST, USA) were incubated with membranes at 4°C overnight. The membranes were cleaned with TBST for 10 min before being incubated with an HRP combined secondary antibody (BioTNT, China) for 1 h at room temp. The membranes were then wiped three times with TBST for 10 min each. Finally, ImageJ software was used to quantify the band intensity after the protein bands had been visualized with ECL.

### Statistical analysis

The data were analyzed using GraphPad Prism eight and were all represented as means ± standard deviation (SD). The differences between the groups were taken into consideration using a one-way analysis of variance (ANOVA). Statistical significance was defined as a *P*-value < 0.05.

## Results

### Effects of notoginsenoside Fc on serum biochemical parameters in APAP-induced AKI mice

**Figure 1:**
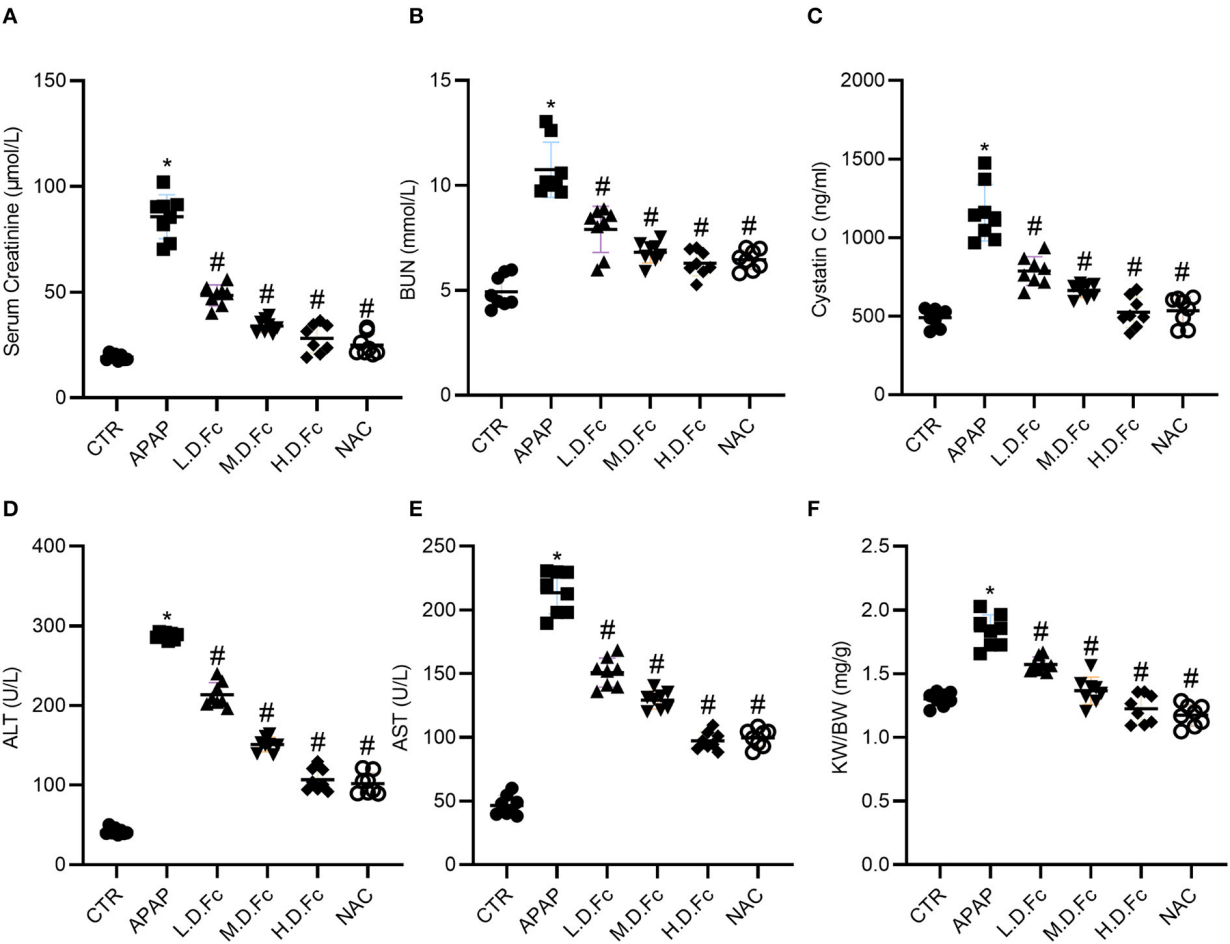
Effects of Notoginsenoside Fc on Biochemical Parameters of Serum Caused by APAP. **(A)** The levels of serum creatinine. **(B)** The levels of BUN. **(C)** The levels of Cystatin C. **(D)** The levels of alanine aminotransferase (ALT). **(E)** The levels of aspartate aminotransferase (AST). **(F)** The renal index (KW/BW). Results were expressed as mean ± SD (*n* = 8). *P < 0.05 vs. normal control mice. ^#^*P* < 0.05 vs. APAP-induced AKI mice.

The effects of Fc on the biochemical parameters of serum were assessed in mice of C57BL/6 strain caused by APAP. In APAP-induced AKI model, the mice showed noticeable elevation of Scr, BUN and Cystatin C when compared with the control group at 36 h after administration of APAP. Pretreatment with different concentrations of Fc significantly decreased the serum level of Scr, BUN and Cystatin C in APAP-induced AKI mice when compared with the APAP control group (Figures 1A–C). And there was a significant increase in the serum levels of ALT and AST when compared with the control group, suggesting that APAP caused toxicity to the liver. However, different concentrations of Fc significantly decreased the serum level of ALT and AST (Figures 1D,E). These results indicated Fc could improve the kidney and liver function in APAP-induced AKI mice. Additionally, Fc pretreatment dramatically reduced the kidney weight/ body weight in APAP-induced AKI mice.

### Effects of notoginsenoside Fc on histologic alterations in APAP-induced AKI mice

**Figure 2:**
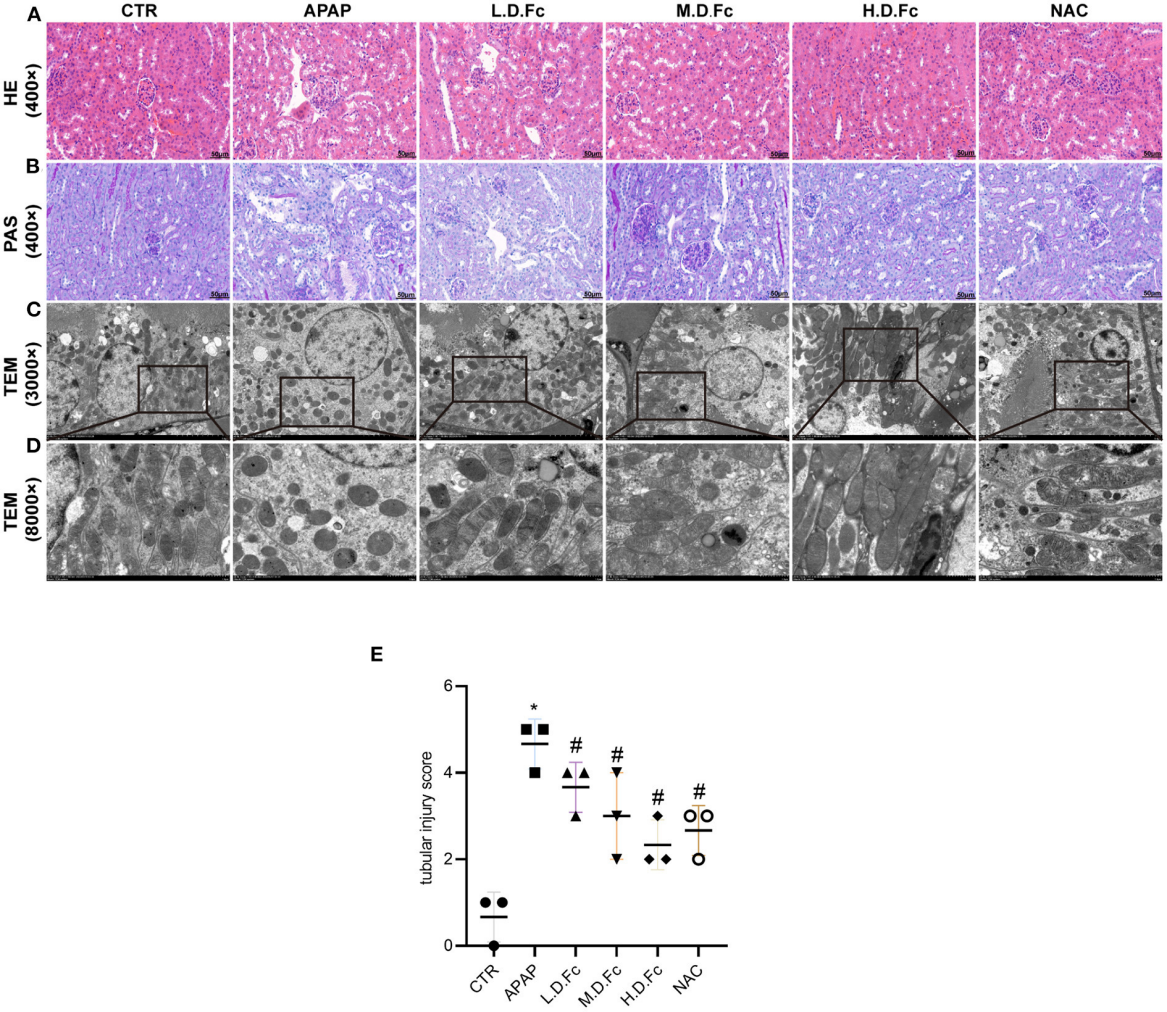
Notoginsenoside Fc improved renal histopathology and mitochondrial morphology abnormalities in renal tubular cells. **(A,B)** Renal histology evaluations were performed with HE and PAS staining (original magnification × 400, bars = 50 μm). **(C)** Mitochondria morphology alterations in renal tubular cells were observed by TEM (original magnification × 3,000, bars = 5 μm). **(D)** High-magnification TEM micrographs of mitochondrial ultrastructure (original magnification × 8,000, bars = 1 μm). **(E)** Tubular injury score. Results were expressed as mean ± SD. *P < 0.05 vs normal control mice. ^#^*P* < 0.05 vs. APAP-induced AKI mice.

Comparing with control group, the mice in APAP group showed clear renal histologic alterations (Figures 2A,B). Particularly, the renal tubular architecture had the most extreme and dramatic changes, including significant dilatation of the renal tubules and renal tubular vacuolar degeneration (Figures 2A,B). Fc pretreatment resulted in significantly reduced histologic characteristics of these renal tubular lesions in kidney sections from the APAP groups (Figures 2A,B). Using TEM, changes in mitochondrial morphology were discovered. According to Figures 2C,D, most renal tubular cells in AKI animals had spherical or rod-shaped mitochondria with partially dissolving cristae. These alterations were prevented by Fc or NAC pretreatment.

### Effects of notoginsenoside Fc on renal tubular apoptosis in APAP-induced AKI mice

**Figure 3:**
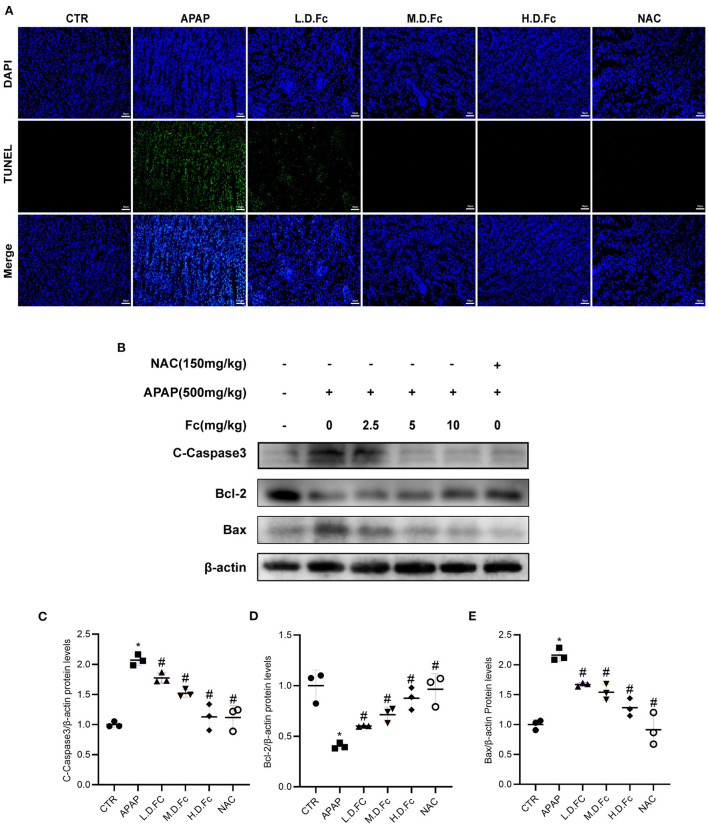
Notoginsenoside Fc attenuated renal tubular cells apoptosis in AKI mice. **(A)** Representative double immunofluorescence labeling, including the TUNEL assay and DAPI on frozen kidney sections. **(B–E)** Representative images of Bcl-2, Bax and C-Caspase3 protein expression and semiquantitative analyses in different groups. Results were expressed as mean ± SD. *P < 0.05 vs. normal control mice. ^#^*P* < 0.05 vs. APAP-induced AKI mice.

As shown in Figure 3, the tubular compartment of the kidneys of AKI mice included a higher density of TUNEL-positive cells. However, in APAP-induced AKI animals, Fc or NAC could greatly reduce the renal tubular cells' apoptosis (Figure 3A). Additionally, we used a western blot to find the presence of apoptosis-related proteins in the renal tissues of AKI mice. Bcl-2, a protein thought to be antiapoptotic, was dramatically decreased in AKI mice. Additionally, AKI kidneys showed increased levels of proapoptotic proteins such as Bax and C-Caspase3. All of these alterations were ameliorated by Fc pretreatment (Figures 3B–E).

### Effects of notoginsenoside Fc on regulating SIRT3/SOD2 signaling pathway in APAP-induced AKI mice

**Figure 4:**
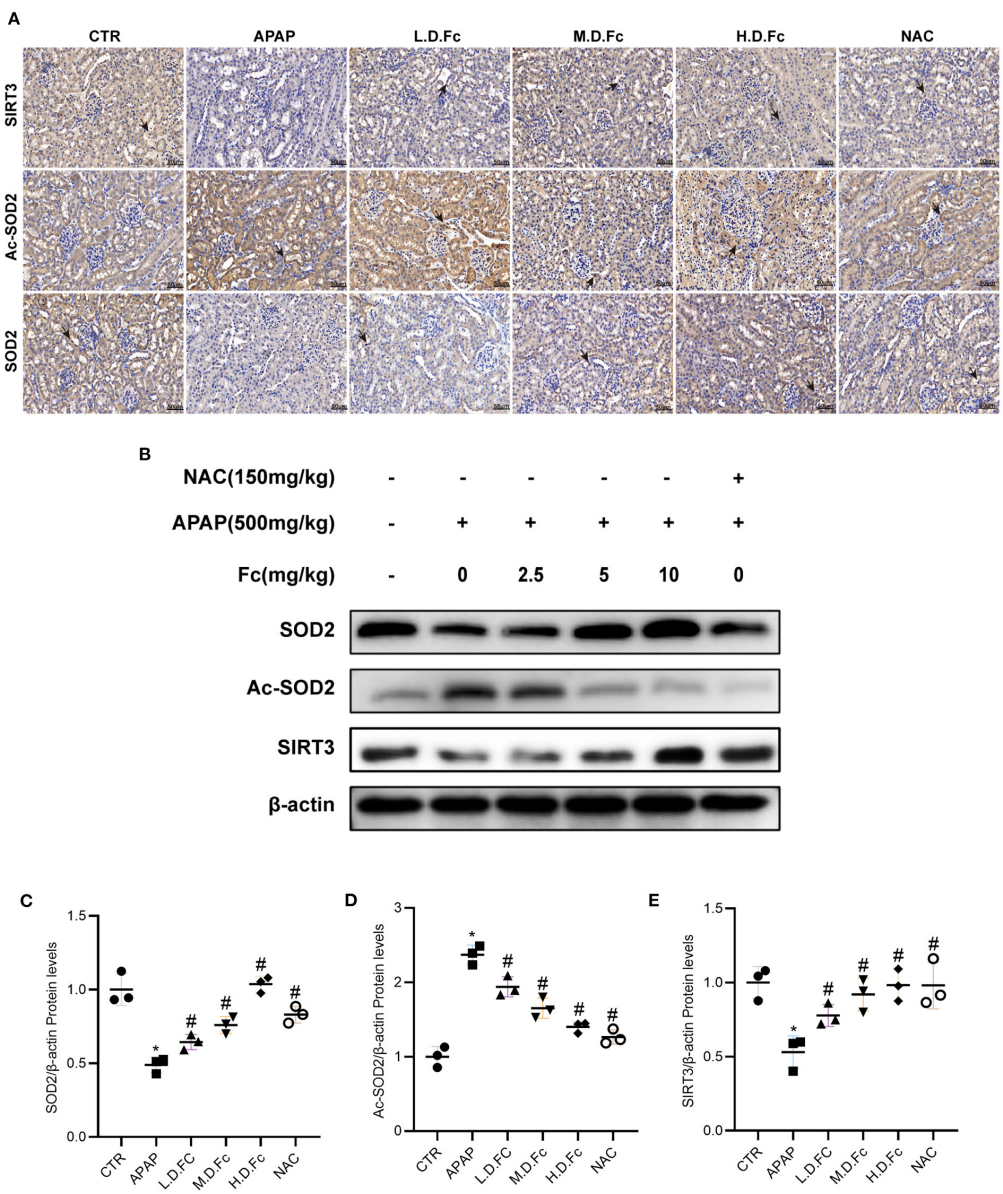
Notoginsenoside Fc restored the expression of oxidative stress-associated proteins in AKI mice. **(A)** Representative photographs of immunohistochemistry staining of SIRT3, Ac-SOD2 and SOD2 (original magnification × 400, bars = 50 μm). **(B–E)** Representative Western blot images of SIRT3, Ac-SOD2 and SOD2 protein expression and semiquantitative analyses. Results were shown as mean ± SD. *P < 0.05 vs normal control mice. ^#^*P* < 0.05 vs. APAP-induced AKI mice.

Western blot and immunohistochemical labeling were used to examine the expression of SIRT3 and SOD2 in mitochondria. Reduced protein expression of SIRT3, SOD2 and elevated Ac-SOD2 in the kidneys of AKI mice. However, Fc or NAC treatment restored protein levels of SIRT3, SOD2, and Ac-SOD2 in AKI mice (Figures 4A–E). These findings suggested that Fc may exhibit its renoprotective properties partly by regulating SIRT3/SOD2 pathyway.

### Effects of notoginsenoside Fc on apoptosis of APAP-induced HK-2 cells

**Figure 5:**
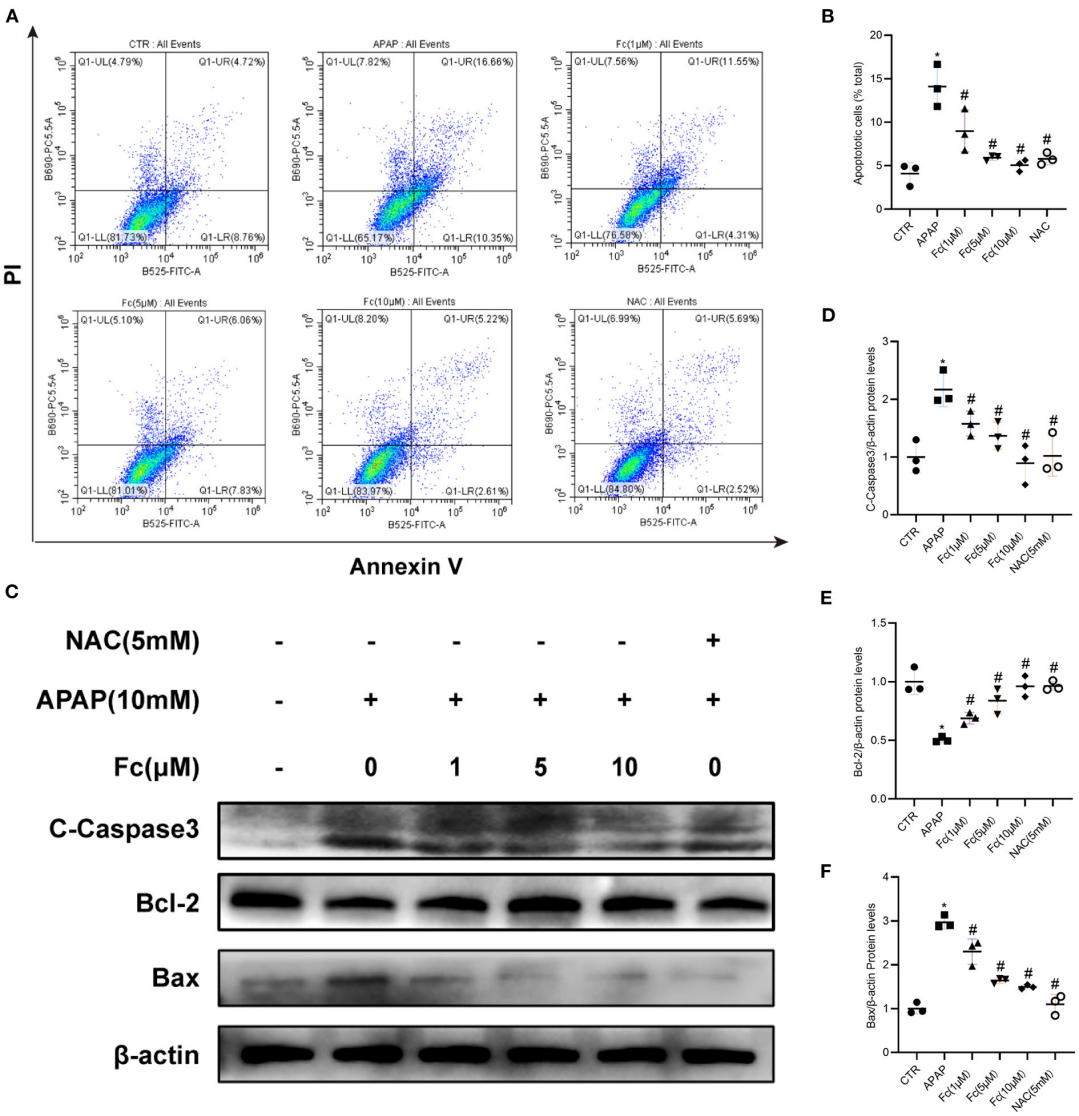
Notoginsenoside Fc reduced the apoptosis of HK-2 cells under APAP exposure. **(A,B)** Representative images of flow cytometry and quantitative analysis of apoptosis rate in HK-2 cells. n = 3. **(C–F)** Representative images of Bcl-2, Bax and C-Caspase3 protein expression and semiquantitative analyses in different groups. Results were expressed as mean ± SD. *P < 0.05 vs control group. ^#^*P* < 0.05 vs. APAP group.

After receiving APAP treatment for 24 h, HK-2 cells experienced a noticeably higher rate of apoptosis. However, both 1, 5, 10 μM Fc and NAC could strikingly reduce HK-2 cells apoptosis (Figures 5A,B). Apoptosis-related proteins were also examined in HK-2 cells. APAP treatment significantly reduced the expression of Bcl-2 and increased the expression of Bax and C-Caspase3 in HK-2 cells. Apoptosis was partially restored by Fc in HK-2 cells under APAP conditions (Figures 5C–F). These findings indicated that Fc could protect HK-2 cells from apoptosis.

### Effects of notoginsenoside Fc on intracellular oxidative stress and mitochondrial dysfunction in APAP-induced HK-2 cells

**Figure 6:**
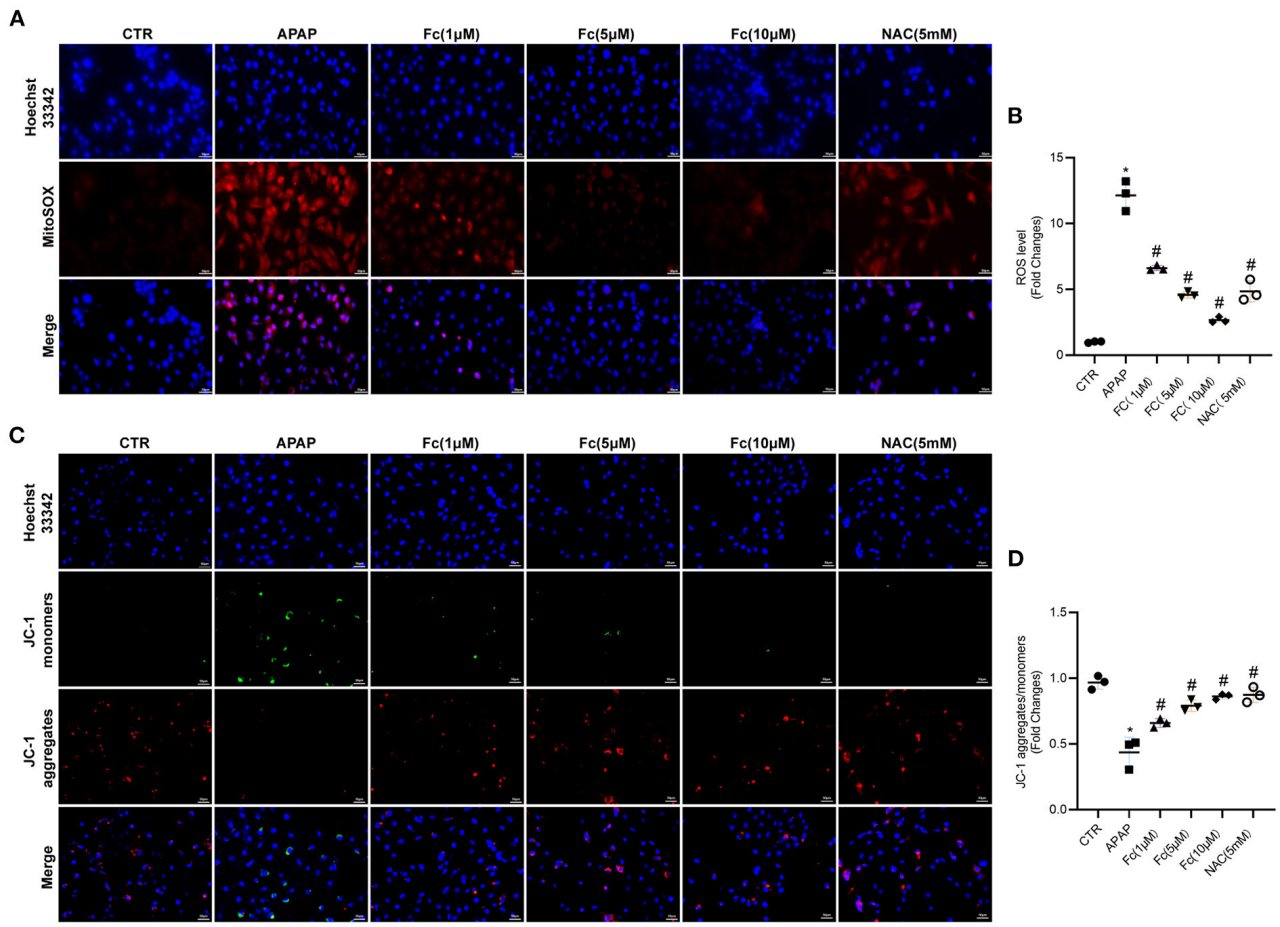
Effects of Notoginsenoside Fc on the intracellular oxidative stress and mitochondrial dysfunction triggered by APAP in HK-2 cells. **(A,B)** Representative images and semiquantitative analysis of mitochondrial ROS production in HK-2 cells (original magnification × 200, bars = 50 μm). **(C,D)** Representative images and semiquantitative analysis of mitochondrial membrane potential levels in HK-2 cells (original magnification × 200, bars = 50 μm). Results were expressed as mean ± SD. *P < 0.05 vs. control group. ^#^*P* < 0.05 vs. APAP group.

The aim was to assess whether Fc could attenuate APAP-induced cell damage by limiting ROS production *in vitro*. Fc was used to pre-treat HK-2 cells before the addition of APAP (10 mM), and NAC was employed as a positive control. We discovered increased superoxide generation in HK-2 cells exposed to an excess of APAP, as shown in Figures 6A,B. Fc had a substantial therapeutic effect that decreased cellular ROS buildup brought on by APAP (Figures 6A,B). A JC-1 kit was used to detect JC-1 aggregates and JC-1 monomers to further support the protective impact of Fc on mitochondria. Our findings demonstrated that an excess of APAP could cause significant loss of mitochondrial membrane potential, whereas Fc could significantly reverse this loss, proving beyond a doubt that Fc's protective effect on APAP-mediated cellular damage resulted from the improvement of mitochondrial dysfunction (Figures 6C,D). Collectively, our results revealed that Fc reduced APAP-induced cytotoxicity by avoiding intracellular oxidative stress and mitochondrial dysfunction.

### Effects of notoginsenoside Fc on regulating SIRT3/SOD2 signaling pathway in APAP-induced HK-2 cells

**Figure 7:**
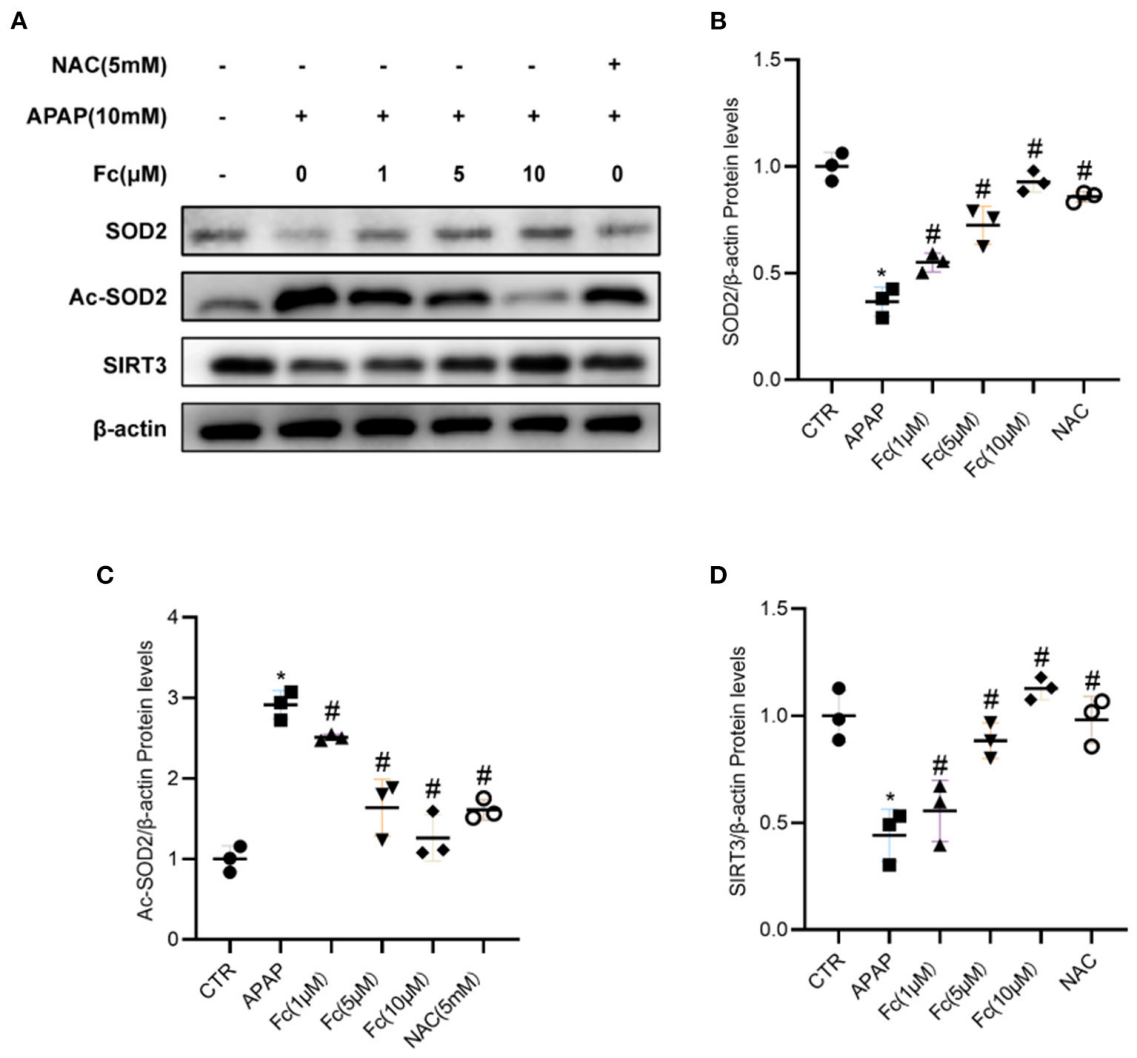
Notoginsenoside Fc restored the expression of SIRT3/SOD2. **(A–D)** Western blot analyses and semiquantitative analyses of expression of SIRT3 and SOD2. Results were expressed as mean ± SD. *P < 0.05 vs. control group. ^#^*P* < 0.05 vs. APAP group.

Next, we looked into how Fc affected the expression of proteins related to mitochondrial oxidative stress. In HK-2 cells, the expression of SIRT3 and SOD2 was also discovered. According to Western blotting data, the expression of SIRT3 and SOD2 was all dramatically reduced by APAP and Ac-SOD2 expression was elevated, but these abnormalities were partially reversed by Fc (Figures 7A–D). Thus, APAP-mediated mitochondrial oxidative stress was partially prevented by Fc regulation of the SIRT3/SOD2 pathway.

## Discussion

It is recognized that AKI is very common in all clinical departments, with a high morbidity and death rate, particularly in ICU. Drug-induced AKI has been considered to be a major barrier in drug development and clinical treatment (17, 18). A common antipyretic analgesic is APAP. An appropriate dosage of APAP reduces pain and temperature, but an overdose can result in AKI (19). It is known that severe AKI requires renal replacement therapy and is associated with a high mortality rate (18). Thus, there is an urgent need to development new drugs for preventing and treating APAP-induced AKI. In this study, we found that Fc pretreatment ameliorated kidney injury as measured by biochemical markers in APAP-induced AKI model. Fc pretreatment decreased the serum level of Scr, BUN, and Cystatin C in APAP-induced AKI mice. Based on histological examinations, Fc significantly ameliorated APAP-induced AKI. Moreover, Fc inhibited the APAP-induced apoptosis of renal tubular cells *in vivo* and *in vitro*. We also found that treatment with Fc for 6 days after a single dose of APAP decreased BUN, Scr and Cystatin C levels in APAP-induced AKI mice (data shown in Supplementary Figure S1). These results indicated the therapeutic efficacy of Fc administration after the kidney injury in APAP-induced AKI. Thus, Fc might be developed to a novel drug candidate for prevention and treatment of AKI.

Models of APAP-induced AKI existed in humans (20) and animals (21). Renal tubular cell pathologic alterations, such as cytoplasmic vacuolar changes and cell death/necrosis, have been identified as a distinctive pattern of proximal tubular injury caused by APAP. We discovered that APAP can activate C-Caspase3, leading to death in cultured HK-2 cells. Several kinds of research have already demonstrated that APAP can directly elicit cytotoxic effects or caspase-dependent apoptosis in renal tubular cells. Recent research revealed that both oxidative stress and inflammation played important role in APAP-induced kindey injury (22–24). Mitochondrial dysfunction resulting from APAP induced the production of ROS, contributing to tubular cell damage and apoptosis (25). However, the precise mechanisms remain elusive. Mitochondria are intricate intracellular organelles that serve critical physiological roles. Mitochondria are tightly packed inside renal tubules. It is believed that a major mechanism underlying the development of AKI is mitochondrial dysfunction (26). Excessive suppression of mitochondrial activity under physiological settings may be harmful to cells and organs. However, oxidative stress and inflammation may be brought on by mitochondrial damage under pathological circumstances. To lessen the damage to the organ, a sufficient regulation of the activity of the damaged mitochondria could limit the generation of ROS and other harmful chemicals. Although the mechanism is not fully known, Inorganic antioxidants, such as glutathione, have been reduced in mitochondria, resulting in an increase in ROS production (27, 28). As C-Caspase3 activity rises and renal cells undergo apoptosis, APAP causes oxidative injury to mitochondrial proteins and lipids (29). These findings implied that ROS, oxidant defense, mitochondria, Bax, Caspases, and APAP-induced nephrotoxicity are all possible targets for enhancing clinical outcomes. Numerous investigations have shown that APAP can cause nephrotoxicity, which could cause rapid renal failure and mortality in experimental animals (30–33). Previous studies indicated that oxidative stress plays an important role in the onset of APAP-induced AKI and ROS participated in cell apoptosis (34, 35). Therefore, oxidative stress and mitochondrial dysfunction may contribute to the podocyte detachment, which represents a important mechanism leading to AKI. The promise of this novel insight is the development of new and effective interventions for prevention and treatment of AKI. To reveal the mechanisms underlying the beneficial role of Fc, we investigated the effects of Fc on apoptosis and mitochondrial dysfunction in APAP-induced AKI mice. We found that Fc attenuated renal tubular cells apoptosis and restored expression of apoptosis-related proteins such as Bax, Bcl-2, and C-caspase3. *In vitro* studies demonstrated that Fc ameliorated the loss of mitochondrial membrane potential and decreased the formation of mitochondrial superoxide. Taken together, the regulatory effects of Fc on mitochondrial dysfunction are likely to be accountable for its action of protecting against renal tubular cells apoptosis.

HK-2 cells were used to investigate the protective effects of interleukin (IL)-22 on APAP-induced renal injury and the underlying mechamisms in previous study (36). Thus, we also used HK-2 cells to study the beneficial effects Fc on APAP-induced kidney damage.

The histone deacetylase SIRT3, which is mostly found in mitochondria and is strongly expressed in the kidney, is a highly conserved NAD+-dependent enzyme (37). SIRT3 is essential for preserving mitochondrial activity. According to one study, it deacetylated important lysine residues to serve as the main deacetylase for mitochondrial target proteins (38). In one of our earlier investigations, it was shown that SIRT3 prevents chronic kidney damage by deacetylating KLF15 (39). SIRT3 enhanced mitochondrial dynamics to guard against AKI, according to Morigi et al. (40). According to a different study, SIRT3 dropped cisplatin-induced AKI's oxidative stress, mitochondrial dysfunction, and tubular epithelial cell apoptosis (41). A recent study demonstrated that SIRT3 deficiency aggravated contrast-induced acute kidney injury (42).

In addition, Fc could prevent this APAP-induced activation of C-Caspase3 and Bax in HK-2 cells directly, reversing the expression of SIRT3/SOD2 in the process. We also found that Fc could significantly boost SIRT3/SOD2 expression in the kidneys *in vivo*. These outcomes suggested that native Fc may have a protective impact on renal tubular cells through the SIRT3/SOD2 signaling pathways *in vitro* and *in vivo*; consequently, we theorized that Fc spontaneously restrained renal tubular cell apoptosis brought on by APAP, blocking the renal dysfunction brought on by APAP. Pathogenesis of AKI is also caused by apoptosis (43, 44). In our research, we discovered that APAP therapy in mice kidney tissue increased the frequency of TUNEL-positive cells in renal tubules while decreasing the expression of C-Caspase3, Bax, and Bcl-2. Fc treatment also significantly resisted apoptosis. In this study, Fc also partly restored the protein expression of Bax, Bcl-2, C-Caspase3, SIRT3, and SOD2 in HK-2 cells exposure to APAP. In summary, Fc might reduce renal tubular injury and mitochondrial dysfunction in AKI partly through the regulation of SIRT3/SOD2 pathway. These findings might contribute to the development of a new therapy for AKI.

Moerover, NAC has a narrow therapeutic window and potential side effects. NAC may cause adverse effects, including vomiting, rash, urticaria and even anaphylactic reactions (45). Therefore, it is desirable to find a new treatment with fewer side effects and more potency than NAC. Our results indicated the therapeutic and preventive efficacy of Fc in APAP-induced AKI. We used HE and PAS staining on the serial kidney sections and also detected the levels of ALT, AST, BUN, Scr and Cystatin C to evaluate the safety of Fc in mice. Our results demonstrated that 2.5, 5 and 10 mg/kg of Fc did not cause apparent toxicity to the kidney as shown in HE and PAS staining about the injury of renal histology. Fc also significantly decreased the level of ALT, AST, BUN, Scr, and Cystatin C in AKI mice. These results indicated that Fc could improve the kidney and liver function in APAP-induced AKI mice. Thus, Fc might be safe for the treatment of APAP-induced AKI. These findings might pave the way to a novel strategy for the prevention and treatment of AKI.

There are some limitations in this study. Firstly, this study did not further explore whether Fc pretreatment could inhibit the apoptosis of renal tubular cells in the SIRT3 knockout APAP-induced AKI model to confirm the mechanisms of Fc through the SIRT3/SOD2 pathway. Secondly, we cannot be certain that the results from the animal model can be translated to humans. Thus, the applicability of Fc in human AKI needs to be investigated in the further study.

In conclusion, our study clearly showed that Fc attenuated tubular injury and mitochondrial dysfunction in AKI mice partly through the regulation of SIRT3/SOD2 pathway. These findings may provide novel therapeutic strategy for AKI. This novel conclusion was highly significant because it might provide a new approach to prevent AKI by targeting inhibition of mitochondrial dysfunction and SIRT3/SOD2 pathway.

## Data availability statement

The raw data supporting the conclusions of this article will be made available by the authors, without undue reservation.

## Ethics statement

The animal study was reviewed and approved by the Animal Ethics Committee of Shanghai Jiao Tong University Affiliated Sixth People's Hospital.

## Author contributions

The research was designed by DG, YX, and HC. MW carried out cell and animal experiments. HC and YG provided ideas and technical assistance. The manuscript was written with assistance from MW, DG, DC, HZ, WZ, YS, QH, XA, BW, ZY, and NW. MW and DG also contributed to the improvement of the figures and language. All authors contributed to the article and approved the final manuscript.
