# Supplementary material for: Notoginsenoside Fc ameliorates renal tubular injury and mitochondrial damage in acetaminophen-induced acute kidney injury partly by regulating SIRT3/SOD2 pathway
Source: Front Med (Lausanne). 2023 Jan 6;9:1055252. doi: 10.3389/fmed.2022.1055252 (PMC9875593; doi:10.3389/fmed.2022.1055252)
Supplement: Supplementary file 1 [file Data_Sheet_1.docx]

Supplementary Material

**Notoginsenoside Fc ameliorates renal tubular injury and mitochondrial damage in acetaminophen-induced acute kidney injury partly by regulating SIRT3/SOD2 pathway**

Miaomiao Wei^1,2†^, Yuancheng Gao^3†^, Dongsheng Cheng^2^, Haiying Zhang^2^,[Wei Zhang](https://pubmed.ncbi.nlm.nih.gov/?term=Zhang+W&cauthor_id=35747383)^4^, Yilan Shen^2^, Qunwei Huang^2^, Xiaoning An^2^, Bing Wang^5^, Zhonghai Yu^5^, Niansong Wang^2^, Hongbo Chen^6^*, Youhua Xu^7^* and Dingkun Gui ^2^^,8^*

^1^College of Fisheries and Life Science, Shanghai Ocean University, Shanghai, People's Republic of China.

^2^Department of Nephrology, Shanghai Sixth People's Hospital Affiliated to Shanghai Jiao Tong University School of Medicine, Shanghai, China.

^3^The Third Affiliated Clinical Medical College, Zhejiang Chinese Medical University, Hangzhou, China.

^4^Department of Nephrology, Shanghai Yangpu Hospital of Traditional Chinese Medicine, Shanghai, China.

^5^Department of Traditional Chinese Medicine, Shanghai Sixth People’s Hospital Affiliated to Shanghai Jiao Tong University School of Medicine, Shanghai, China.

^6^Department of Nephrology, The First Affiliated Hospital of Zhejiang Chinese Medical University (Zhejiang Provincial Hospital of Traditional Chinese Medicine), Hangzhou, China.

^7^Faculty of Chinese Medicine, State Key Laboratory of Quality Research in Chinese Medicine, Macau University of Science and Technology, Macao, China

^8^Department of Central Laboratory, Shanghai Sixth People’s Hospital Affiliated to Shanghai Jiao Tong University School of Medicine, Shanghai, China.

*** Correspondence:**

Hongbo Chen

chenhb521@126.com (H.C.)

Youhua Xu

yhxu@must.edu.mo (Y.X.)

Dingkun Gui
dingkungui@alu.fudan.edu.cn (D.G.)

^†^These authors have contributed equally to this work.

**Methods**

**Animal studies**

Six-week-old male C57BL/6 mice, weighing 20 ± 2g, were housed in a specified pathogen-free (SPF) environment. A 12 h/12 h light cycle and temperature control were provided in the housing. After acclimating to the experimental region for seven days,48 male mice were randomly divided into six groups: Control group (CTR, n= 8), APAP group (500 mg/kg/d, intraperitoneally (i.p). n = 8), APAP + Fc group (low-dose Fc, 2.5 mg/kg/d, intragastric administration (i.g). n = 8), APAP + Fc group (medium-dose Fc group, 5 mg/kg/d, i.g. n = 8), APAP + Fc group (high-dose Fc group, 10 mg/kg/d, i.g. n = 8), APAP + NAC group (150 mg/kg body weight, i.p. n = 8). APAP treatment was given on day 1 and Fc or NAC was administrated to mice from day 2 to day 7. An identical volume of normal saline was intraperitoneally given to control mice.

**Renal Function Analysis**

To assess whether Fc improves renal function in APAP-induced acute kidney injury, blood samples were centrifuged at 12,000 rpm for 20 minutes at 4°. Blood supernatants were used to assess serum creatinine (Scr), blood urea nitrogen (BUN), blood aspartate aminotransferase (AST) and alanine aminotransferase (ALT), the above kits were purchased from Nanjing Jiancheng Bioengineering Institute (Nanjing, China). ELISA kit for cystatin C was purchased from BOSTER (Wuhan, China). Kidney weight and body weight were also measured.

**Results**

**Effects of Notoginsenoside Fc on Biochemical Parameters in APAP-induced AKI mice**

Treatment with Fc for 6 days after a single dose of APAP significantly decreased the serum level of Scr, BUN and Cystatin C in APAP-induced AKI mice when compared with the APAP control group (Figure S1A,B,C). Treatment with Fc also reduced the serum levels of ALT and AST when compared with the APAP control group(Figure S1D,E), suggesting that Fc ameliorated toxicity to the liver. In brief, Fc might improve the kidney and liver function in APAP-induced AKI mice. Moreover, Fc treatment reduced the kidney weight/ body weight in APAP-induced AKI mice. These results indicated the therapeutic efficacy of Fc administration after the kidney injury in animal model of APAP-induced AKI.

**Figure S1.** Effects of Notoginsenoside Fc on Biochemical Parameters in APAP-induced AKI mice. **(A)** The levels of serum creatinine. **(B)** The levels of BUN. **(C)** The levels of Cystatin C. **(D)** The levels of alanine aminotransferase (ALT). **(E)** The levels of aspartate aminotransferase (AST). **(F)** The renal index (KW/BW). Results were expressed as mean ± SD (n=8). *P < 0.05 vs normal control mice. ^#^P < 0.05 vs APAP-induced AKI mice.
