# Supplementary figures and images for: Notoginsenoside Fc ameliorates renal tubular injury and mitochondrial damage in acetaminophen-induced acute kidney injury partly by regulating SIRT3/SOD2 pathway
Source: Front Med (Lausanne). 2023 Jan 6;9:1055252. doi: 10.3389/fmed.2022.1055252 (PMC9875593; doi:10.3389/fmed.2022.1055252)

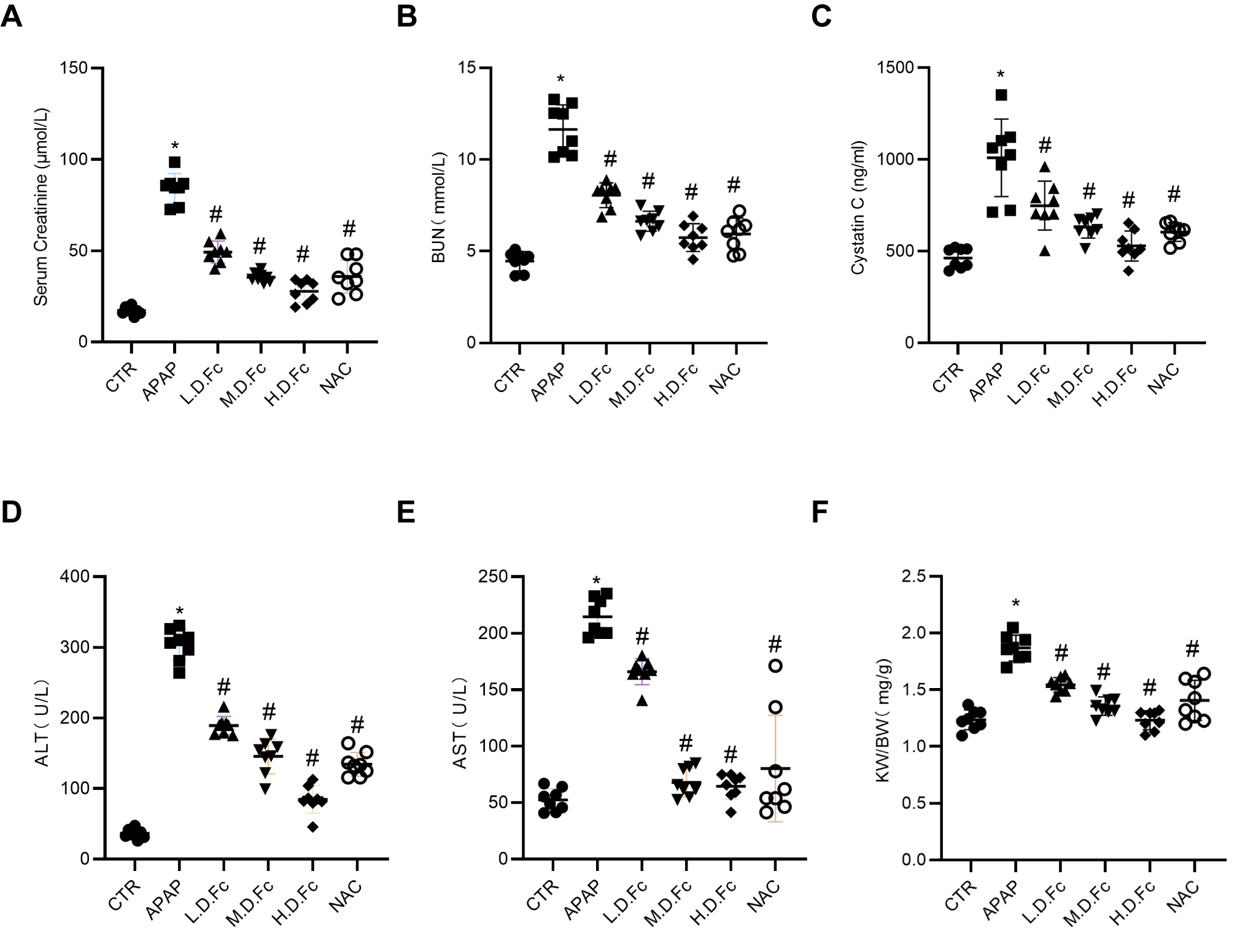

Supplement: Supplementary file 2 [file Image_1.tif]
